# Optimizing a mentorship program from the perspective of academic medicine leadership – a qualitative study

**DOI:** 10.1186/s12909-024-05463-6

**Published:** 2024-05-14

**Authors:** Michael Ren, Dorothy Choi, Chloe Chan, Simrit Rana, Umberin Najeeb, Mireille Norris, Simron Singh, Karen E. A. Burns, Sharon E. Straus, Gillian Hawker, Catherine Yu

**Affiliations:** 1 Unity Health Toronto, St. Michael’s Hospital, Toronto, Ontario Canada; 2 Royal College of Surgeons in Ireland, School of Medicine, Dublin, Ireland; 3 Department of Medicine, University of Toronto Temerty Faculty of Medicine, Toronto, Ontario Canada

**Keywords:** Faculty mentorship, Departmental mentorship program, Social ecological model, Leverage points

## Abstract

**Background:**

Effective mentorship is an important contributor to academic success. Given the critical role of leadership in fostering mentorship, this study sought to explore the perspectives of departmental leadership regarding 1) current departmental mentorship processes; and 2) crucial components of a mentorship program that would enhance the effectiveness of mentorship.

**Methods:**

Department Division Directors (DDDs), Vice-Chairs, and Mentorship Facilitators from the Department of Medicine at the University of Toronto Temerty Faculty of Medicine were interviewed between April and December 2021 using a semi-structured guide. Interviews were audio-recorded and transcribed verbatim, then coded. Analysis occurred in 2 steps: 1) codes were organized to identify emergent themes; then 2) the Social Ecological Model (SEM) was applied to interpret the findings.

**Results:**

Nineteen interviews (14 DDDs, 3 Vice-Chairs, and 2 Mentorship Facilitator) were completed. Analysis revealed three themes: (1) a culture of mentorship permeated the department as evidenced by rigorous mentorship processes, divisional mentorship innovations, and faculty that were keen to mentor; (2) barriers to the establishment of effective mentoring relationships existed at 3 levels: departmental, interpersonal (mentee-mentor relationships), and mentee; and (3) strengthening the culture of mentorship could entail scaling up pre-existing mentorship processes and promoting faculty engagement. Application of SEM highlighted critical program features and determined that two components of interventions (creating tools to measure mentorship outcomes and systems for mentor recognition) were potential enablers of success.

**Conclusions:**

Establishing ‘mentorship outcome measures’ can incentivize and maintain relationships. By tangibly delineating departmental expectations for mentorship and creating systems that recognize mentors, these measures can contribute to a culture of mentorship.

**Supplementary Information:**

The online version contains supplementary material available at 10.1186/s12909-024-05463-6.

## Background

The increasingly challenging landscape of academic medicine has resulted in rising faculty departure and burnout rates [1–3]. Faculty are facing more regulatory demands and greater pressures to secure grants and increase clinical activities to maintain revenue [2].

Mentorship is a potential strategy to help faculty overcome these challenges and improve faculty retention and promotion [2]. Mentorship involves an individual supporting another individual professionally and personally [4], and it has been associated with improved career satisfaction, successful promotion, a greater sense of support, and higher faculty retention [5, 6].

The success of a mentorship program is dependent on multiple factors [7]. First, certain traits of mentors and mentees can predispose them to effective mentorship, such as the mentor’s ability to create a safe environment and the mentee’s willingness to learn [8]. An academic institution can also facilitate mentorship success by having departments incentivize faculty to engage in mentorship, creating gender-matched relationships, and initiating relationships early in the mentee’s career [9]. Other valuable program components include a mentorship curriculum, a mentorship pairing mechanism, methods to evaluate the program, and compensation for participants [7].

Prior work has focused on the perspectives of mentees and mentors [10]. However, the mentee-mentor relationship does not exist in a vacuum; rather it exists within the contexts of the department, the institution and broadly, academic medicine. As such, the relationship must be viewed as a complex system [11], with stakeholders at multiple levels being accounted for. Hence, a macroscopic understanding of faculty mentorship is warranted, which can be achieved by understanding the perspectives of departmental leadership on mentorship using the lens of the Social Ecological Model (SEM). SEM is a theory that suggests the processes and phenomena that people experience, such as their development and relationships, are influenced not only by factors at the individual level (ex. personal characteristics), but also by their external environment (ex. cultural norms) [12–14].

Departmental leaders are crucial in creating and maintaining a mentorship infrastructure [15] and thus their engagement is critical for mentorship success. Additionally, they have an encompassing perspective of the program, the mentoring relationships within, and can elucidate the mentorship needs at various career stages since many are experienced faculty. Thus, departmental leadership can provide insights about faculty mentorship at multiple organizational/institutional levels, and as posited by SEM, such systems (i.e. mentorship) are influenced by interacting factors that exist from the individual level to the environmental level [12]. In this model, these factors can be organized into five different levels: microsystems, mesosystems, exosystems, macrosystems, and chronosystems. The microsystems are the relationships themselves, the mesosystems focus on the interactions between relationships, the exosystems factors in the external settings, the macrosystems encompass the culture of the micro-, meso-, and exosystems, and the chronosystems account for changes across time [12–14]. SEM has been used to understand complex systems, such as healthcare, in order to develop interventions to address systemic problems [16], and it can be applied to mentorship as well [13]. The model emphasizes that the mentoring relationship exists within and is influenced by these levels and that success depends on acknowledging the interconnections across those contexts. Accordingly, we sought to explore the perspectives of departmental leadership regarding 1) current departmental mentorship processes; and 2) crucial components of a mentorship program that would enhance the effectiveness of mentorship.

## Methods

### Study design

This was a qualitative study using individual interviews and a descriptive analysis [17]. We reported our study according to the Standards for Reporting Qualitative Research checklist (Additional file 1). The study was approved by the University of Toronto Research Ethics Board.

### Setting and participants

The Department of Medicine at the University of Toronto Temerty Faculty of Medicine has over 900 full-time faculty members and 20 divisions, the largest medicine department in Canada. The department leadership is composed of Department Division Directors (DDDs), and Vice-Chairs of Research, Quality and Innovation, Education and Culture and Inclusion. These leaders help advance the department’s patient care, research, quality and innovation, and education goals.

### Description of the faculty mentorship program

A multifaceted mentorship program was created in 2015 to: 1) Promote effective faculty mentorships; 2) Provide easy access to effective mentorship; and 3) Acknowledge the importance of mentorship.

At the time of recruitment, all newly appointed faculty members identify an appropriate formal mentor through a joint discussion with their DDD and physician-in-chief. This involves the mentee identifying their needs and receiving input from departmental leaders. All faculty can request new or additional formal mentors by reaching out to their DDD and going through a similar process. The formal mentor cannot be a faculty member to whom the mentee reports to (Ex. their DDD). After a formal mentor is identified, the mentee and the mentor create a five-year academic plan which is then approved by the departmental leaders.

The program has several key players. First, the DDDs ensure that faculty members have effective mentorship; this is reviewed annually, wherein they discuss mentorship quality, provide support and feedback and address any issues. Second, the Vice Chairs conduct a 1.5-year check-in to address academic goals, work-life balance, and mentorship satisfaction. Third, divisional Mentorship Facilitators (faculty members selected by the Mentorship Committee after an open call) facilitate connections between mentors and mentees; monitor and provide feedback to the relationship; and provide mentorship support and resources to the division. Finally, expectations for mentors and mentees are reviewed at the mandatory New Faculty orientation session. Mentees are expected to drive the relationship and topics of discussion; which includes meeting with their formal mentors three or four times annually during their first 5 years in order assess their academic progress.

Faculty surveys are conducted every 1–2 years (pause for COVID-19 pandemic) to evaluate mentorship. Further, faculty members’ perception regarding the mentorship program have also been collected. These data will be reported in separate manuscripts as they are not the focus of the current study. Lastly, yearly departmental and divisional awards are presented.

### Recruitment

All DDDs (with Divisions comprised of more than 5 faculty members), Vice-Chairs and Mentorship Facilitators were invited to participate in semi-structured interviews by the mentorship lead (C.Y.) for the Department of Medicine through email.

### Data collection

Interviews were conducted by a trained interviewer (C.Y.) on the Zoom platform (Zoom Video Communications Inc., 2016) using a semi-structured interview guide that was created de novo to explore current mentorship processes and potential opportunities for enhancement; it was tested on team members and refined iteratively (Additional file 2). Interviews were audio recorded, transcribed verbatim, and deidentified.

### Data analysis and role of theory

First, each transcript was coded by at least two analysts using a descriptive analytic approach [17]. The codes were refined and discrepancies were resolved during team meetings. Each team member had different research backgrounds, enabling investigator triangulation as members provided unique interpretations [18]. Field notes were made to promote reflexivity as they highlighted additional insights [19]. Data saturation was achieved when redundancy occurred during coding. We developed a preliminary coding framework using inductive content analysis [17], then identified themes important for program development. NVivo version 12 (QSR International, Melbourne, Australia) was used [20].

Second, we applied SEM to interpret the findings. From the coding framework, we identified important characteristics of the mentorship program and categorized them into these systems: relationship (*micro*), divisional/departmental (*exo*), and academic medicine (*macro*). We then identified similarities among these characteristics and cross-categorized these program characteristics into six components: expectations, mentor identification, relationship maintenance, mentor recognition, faculty development, and measurement. Finally, we reviewed each characteristic vis-a-vis its ability to make a change at multiple levels, and labeled such characteristics as “leverage points” [21].

## Results

### Characteristics of participants

Fourteen DDDs, three Vice-Chairs and two faculty Mentorship Facilitator-Leads were individually interviewed. Characteristics of the participants are listed in Table 1.

**Table 1:** Gender and Race of Interview Participants, 2022

**Gender** (n)	
Female	7
Male	8
Did not disclose	4
**Race** (n)	
Black	0
East Asian	4
South Asian	2
Southeast Asian	0
Middle Eastern	1
White	8
Mixed	0
Did not disclose	4

#### Theme 1: A culture of mentorship permeates the entire department as evidenced by rigorous mentorship processes, divisional mentorship innovations, and faculty that are keen to mentor

Leaders remarked that departmental mentorship processes were diligently implemented. Faculty leadership prioritized and worked with new faculty to identify appropriate mentors (Table 2). After a match is made, leadership documented the relationship in the academic planning document, and felt that formalization of the relationship enabled greater mentee engagement and mentor responsibility.

*“You know the nice thing about having a formal mentor is…it gives licence to the mentee to be able to push a little bit more than they would if they didn’t have that formal relationship.” (DDD 5).*

Departmental leadership also noted the formal 1.5-year faculty check-ins seemed to improve outcomes of continuing faculty appointment reviews.

*“I have not seen nearly as much trouble at CFAR…I do think that one and a half year check-ins…and the higher level of attention that has been paid to getting younger trainees mentorship…you can see it paying off…I think that people who are faculty members who are five years or less seem much more prepared than I have seen previously.” (DDD 8).*

Secondly, DDDs reported prioritizing mentorship at the divisional level. Some divisions developed methods to document mentoring relationships for example by creating spreadsheets (Table 2). Others initiated their own professional development activities, such as facilitated peer mentoring groups and a life-coach lecture series.

*“[T]he early career folks, I think I have a good handle on since I started having sessions with the junior faculty. Between them and myself. To allow them to be able to feel safe to articulate their feelings…That has been very helpful for the junior faculty because among themselves they realize they all face the same challenges and issues.” (DDD 12).*

Mentorship awards also existed in many divisions (Table 2).

**Table 2:** Representative Quotes from Departmental Leadership Regarding the Mentorship Culture in the Department, 2021

Subthemes	Quotes
***Departmental mentorship processes***	
Mentor-mentee pairing process	*“*When everyone comes on, we make sure they have a mentor and that is a big focus of discussion at the time of hiring. So, there’s two things that we want to get as right as possible. One thing is their academic planning document…[t]he second is identifying a mentor.*”* (DDD 1)
Formalization of mentoring relationship	“I think the new people value it because they want to pass their CFAR, they want to make sure they’re doing everything right and I think they appreciate having someone assigned whose responsible for them.” (DDD 10)
***Divisional mentorship innovations***	
Divisional-level documentation	“A lot of the focus was trying to set up an organized mentorship program where there was stuff on paper where we had a way of tracking if everybody had mentors and just keeping track of trying to quantify, or maybe even get some qualitative stuff about how mentorship was working.” (DDD 6) “I have a spreadsheet for all of the faculty in my division where I track their various academics, so when they were appointed, when they were promoted, when they’re up for promotions or CFAR, and who their mentor is” (DDD 10)
Group activities for faculty	“I’m going to start some lecture guidance programs next year for all faculty so they can help them figure out what they want to do, how to conduct their life. We’ll get external speakers either within the university or not.” (DDD 9) “I think the junior folks probably need the most mentoring, the more junior you are. And that can be by having a senior mentor, but I know in our division, probably the most effective thing was having a peer group mentorship. So, there was one very junior mentorship group that started out a few years ago. And they got to more senior like mid-level and then a new group started and they’re maturing… it started organically, they decided to be together. And that’s totally fine, that’s probably the best way to start it actually” (DDD 1)
Divisional mentorship award	“So, we’ve created a mentorship award which we’ve named after… Dr. [Name]. So, in order to lift up its prestige, we’ve made it a named award which every year since Dr. [Name] is still with us, he personally gives that award. So, we’ve tried to raise this trait of mentorship. Make it a real recognizable rewarding venture, given the time and effort it takes to be a good mentor.” (DDD 12)

Lastly, DDDs found that many faculty members are eager to be mentors.

*“I think people are happy to do it to support the early career faculty. You know its kind of an honor to be asked to be a mentor for someone. People don’t seem to mind being asked.” (DDD 10).*

Overall, a culture of mentorship exists in the department, and is sustained by faculty who value mentorship.

#### Theme 2: Barriers to the establishment of effective mentoring relationships exist at 3 levels; departmental, interpersonal (mentee-mentor relationships), and mentee

Departmental barriers to mentorship revolved around policies, expectations, and resources. For example, DDDs were confused about the duties of the Divisional Mentorship Facilitators, leading to implementation issues and logistical inconsistencies.

*“I feel like these mentorship facilitators at the divisional level were assigned, but I don’t know if they really ever had formal responsibilities assigned or like if they interacted with people …I just feel like my mentorship lead would like a little bit more of a lead from the DOM in terms of responsibilities and expectations” (DDD 5).*

Developing metrics to track mentorship activities and evaluate mentorship quality was challenging and controversial (Table 3). Some participants suggested using metrics that evaluate internal measures like satisfaction while others recommended tangible outputs such as academic productivity. However, some participants were against using either metric, opining that internal measures may not reflect long-term impact and that academic productivity is not necessarily linked to effective mentorship.

*“I’m not a big believer in sort of career pathway and career happiness indexes. I’m not sure that’s a very good way to measure very much other than happiness, and happiness is pretty ephemeral.” (DDD 14).*

*“[T]he tangible outputs of academic success also become some of the benefits of a successful mentorship. But that can’t be the measure.” (DDD 7).*

Lastly, some participants perceived a paucity of available mentors due to faculty turnover and difficulties identifying people outside of one’s network.

*“[P]art of the problem is knowing all the players. There are good people at other sites, there are good people at this site that I don’t know! [B] eing too much in our own sort of little sphere and then you don’t pick up on who are the great…*” *(DDD 7).*

Participants also identified relationship-level barriers, such as finding time to cultivate a relationship (Table 3) and conflicts of interest.

*“I find for a lot of mentors…[t]hey think they’re mentors but they’re actually supervisors … they don’t always recognize that this is the conflict of interest in being a supervisor and a mentor.” (DDD 11).*

Participants also reported mentor-related barriers, such as disenfranchisement (unengaged mentees) and lack of institutional recognition.

*“I think it has to be given more value from the academic institutions… people are already really pushed I think for a whole variety of different reasons … so I think people feel that there’s an expectation but no particular reward.” (DDD 14).*

Other mentor-related barriers include mentors feeling ill-prepared, as well as unclear roles and responsibilities (Table 3).

Lastly, participants identified mentee-specific barriers, such as undervaluing mentorship. Participants also expressed that it can be challenging for mentees to find mentors that met specific needs. Institutional policies such as the ineligibility of faculty in leadership positions to be the formal mentors of faculty members that they directly oversee, limits the mentor pool.

*“sometimes the people who are the most logical mentors are people who are technically not allowed to be the formal mentor because they are… they have a leadership position that makes them ineligible.” (DDD 10).*

Mentee timidness and a perception of mentor unapproachability may also dissuade mentees from reaching out to potential mentors. Furthermore, if mentees expect immediate results in their professional growth, they may devalue the relationship if results are slow to manifest (Table 3).

**Table 3:** Representative Quotes from Departmental Leadership Regarding the Barriers to Mentorship, 2021

Subthemes	Quotes
***Departmental-level barriers***	
Unclear expectations regarding designated divisional mentorship facilitators	“I was very surprised when something came up recently … saying that there was departmental funding for a mentorship person, because that wasn’t what I had been told.” (DDD 14)
Mentorship metrics	“I think there’d be some level of quality or satisfaction piece, that’s the faculty feeling like they’re getting the mentorship that they need.” (DDD 2) “Like I don’t know …if someone didn’t pass their CFAR or didn’t get promoted, I don’t know if I would …if it would fair to hold the mentor accountable for that.” (DDD 10)
Number of available mentors	“[M]entors may retire so what do you in terms of mentorship turnover, when your mentor retires and is not available anymore?” (DDD 5)
***Relationship-level barriers***	
Issues with time	“I think it’s that cultivation of time and commitment that is required, which we just don’t have.” (DDD 9) “[P]eople are busy and so I think its sometimes hard for people to meet as regularly as they would like to.” (DDD 10)
Feelings of disenfranchisement	“I’ve been assigned to be a mentor for people and they didn’t want it, I tried my best…I would try to offer my input. But if they thought they were doing fine and didn’t want any input then that wasn’t going to go anywhere.” (DDD 1) “I’ve had some mentees when I’ve literally- I’ve been chasing them, right? Like, don’t you think it’s time for us to meet? Like, how are things going? And they’re like, you know, they’re too busy or whatever. So, you know, it’s like, okay. You know, obviously, it’s like… I’m not sure if telling them this is your expectations that it’s gonna make any difference.” (DDD 2)
Lack of mentorship training	“I found when you ask people…they’re a little bit tentative because they feel like they don’t have mentorship training” (DDD 8) “I hate to say this but some of it may be generational…, it’s a different world right now…[the] definition of [a] mentor is somebody who supports somebody’s career and makes sure that it blossoms the way that individual wants it to go…I’m not sure that is shared by everybody, I think there still… “this worked really well for me, and you have to do it.”” (DDD 14)
Unclear expectations	“I’m not sure the mentors really know what’s expected of them, so I think it’s kind of lack of clear expectations around what this person should be doing.” (DDD 2)
***Mentee-level barriers***	
Lack of perceived need for mentorship	“I would say a challenge is to be sure that people understand why and how they could benefit [from mentorship].” (DDD 3) “If on the other hand, they’re saying “I’m doing all the stuff I want to do, I’m happy where I am, I’m doing all the things that are meaningful to me, I don’t see a need to change anything.” I don’t know that a mentorship relationship would be helpful or accepted by them.” (DDD 1)
Difficulty finding a mentor	“[P]eople…, might be too shy or they may not have enough confidence, or they might not [be] sure it’s appropriate.” (DDD 2)
Unrealistic expectations	“I get the impression that some mentees are looking for… spoon-feeding… sometimes people don’t recognize that they’re getting helpful advice until later on… personal growth things don’t happen instantaneously.” (DDD 7)

Overall, multiple factors – at departmental, relationship, and individual levels – can impair effective mentorship.

#### Theme 3: Building upon a strong culture of mentorship involves scaling up pre-existing mentorship processes and having a thoughtful approach towards promoting faculty engagement

Scaling up pre-existing mentorship processes requires a balance between standardizing and individualizing mentorship practices. Departmental leadership emphasized the importance of consistency and standardizing the definition of mentorship, the expectations and responsibilities of departmental leaders, and mentorship resources (Table 4).

*“One of the roles of having department of medicine is to set expectations that are consistent across divisions…when I’m replaced and the new DDD comes in…they don’t have to worry about, “am I under doing it or overdoing it,” this is what the department of medicine’s expectations are.” (DDD 11).*

On the other hand, participants stressed that the department needs to go beyond the superficial requirements of having a program that mandates a formal mentor assignment “on paper” because it fails to address the nuances of individual faculty needs (Table 4). Hence, participants noted that individualization was still required to address unique mentorship needs based on training location, career stage, faculty academic position description, and social factors. First, faculty members who trained outside of the institution are less likely to have a network at the institution and thus may benefit from a formal mentorship program.

*“[D]id they train in the UofT system versus not? So, if you have somebody coming from outside, they might need a much more intensive mentorship, like support, than somebody who’s been around and probably has like an informal network of mentors already.” (DDD 10).*

Second, DDDs stated that each career stage has unique mentorship needs. They believed that early-career faculty members have the *greatest* need for mentorship as there are important professional milestones to achieve. For mid-career faculty, participants perceived that their mentorship needs involve achieving promotion or developing new goals (Table 4). Similarly, late-career faculty mentorship needs were dependent on whether they had new goals or wanted to transition into retirement.

*“There are some senior, more seasoned people who I’m not going to go “hey I know you’ve been at this for 20 years, but I still think you need a mentor.” I would only say that if they have a goal in mind that they want to get to.” (DDD 1).*

Third, faculty leadership reported that faculty academic position descriptions affect mentorship needs. For faculty members focusing on education (clinician teachers and educators), participants felt that they require more assistance in finding a mentor and obtaining academic recognition for promotion. Participants reported that the mentorship needs for clinician scientists and investigators revolved around establishing a research network. Clinicians in quality and innovation (CQI) also require networks; however, participants believed the networking in this field is more nuanced and difficult due to its nascency (Table 4).

*“The clinician investigators and the clinician scientists probably need a lot of mentorship too because they have to make those right research connections so that they’re successful with grants and papers so that’s critical that they have a mentorship group.” (DDD 1).*

Fourthly, faculty leadership perceived ethnicity, gender, or other social identities may impact mentorship needs. Faculty leaders outlined needs that may be unique to women, and they include sponsorship, family planning, and the need for women role models. Perceived benefits of identity concordance include familiarity with experiences and obstacles.

*“[Y]eah mentoring is definitely important for that. Women have said repeatedly that “if we want to promote women early on, we need to have more mid and late-career women showing the example right.” (DDD 3).*

Promoting faculty engagement in mentorship is also crucial to building a strong culture of mentorship and requires a thoughtful approach to its prioritization and incentivization. Participants remarked that the institution can place a greater priority on mentorship through policy changes and creating innovative mentorship resources and initiatives. Participants suggested that systemic changes could involve the department formally tracking mentorship and placing a greater focus on mentorship in the job descriptions of faculty leaders.

*“I do think that the role of the PIC and DDD’s and division heads in mentorship has grown. I think it really has developed. I do think that there’s an opportunity to provide more mentorship.” (DDD 8).*

They also noted that innovative faculty development sessions and resources that teach the knowledge and skills could foster effective mentoring relationships (Table 4). Furthermore, participants mentioned that creating more social events provides mentees with more mentorship opportunities and promote a ‘community of practice’ for mentors. Moreover, faculty leadership believed that providing incentives, such as credits for promotions, mentorship awards, and Maintenance of Certification (MOC) credits, to mentors and mentees can increase mentorship involvement.

*“[M]entorship is now being recognized as a very critical role in any academic institution. And if we don’t recognize and reward it then we fail many faculties who demonstrating strong mentorship. So, I think if we do that in the DoM, it should be evident that it’s a real thing, not something we just talk about, that it counts, and people can attest to that it counted in the promotion. The time they spent doing it well recognized and beneficial.” (DDD 12).*

**Table 4:** Representative Quotes from Departmental Leadership Highlighting Strategies to Building Upon a Pre-Existing Mentorship Culture, 2021

Subthemes	Quotes
***Scaling up pre-existing mentorship processes requires a balance between standardization and individualization***	
Factors that push towards standardization	
• Lack of awareness and usage of mentorship resources	“I’m not sure anybody’s using the mentorship checklist. I have to be honest. I’m not sure I’ve ever used it.” (DDD 2)
Factors that push towards individualization	“it’s almost like an exercise in checking boxes and not necessarily effective or what’s needed… So, you come in as a department or division leader and say okay mentorship is good so that means everyone should have a mentor. We make sure everyone has this spreadsheet filled out with their name, make sure that they meet every 3 months, make sure we measure the mentorship, and everything will be good in the world. I’m not personally sure it really works that way.” (DDD 1) “We say, we have to have a mentorship program, we have to train our mentors, we have to make sure our people meet regularly. I think that’s an exercise in futility and it has to be a lot more nuanced and individualized than that.” (DDD 1)
• Career stage	“I do find the mentorship needs are the greatest in the first few years in somebody’s career…[they] got to get through [their] CFAR like there’s a clear hurdle [they] got to get over to maintain [their] career at this institution” (DDD 5) “[T]here’s 3 ways you can look at it: I haven’t hit the goals that I wanted to, so what do I do now? I’ve hit all the goals that I wanted to, what do I do now? Or you know what, I never thought about this opportunity, but I think I can change direction and move on this opportunity…So, who do you go to for all of that?” (DDD 9) “I do think they need somebody who they can reach out to for issues around closing practices and sort of transferring the reins, succession planning.” (DDD 14)
• Job description	“I think where it is maybe a little bit harder to develop an organic relationship are the non-research-based clinician teachers, that sort of individual sometimes needs more pairing.” (DDD 7) “I think that’s a big a thing. Trying to figure out how to get clinician-teachers to get that academic recognition and so like the mentors that have been through that and been successfully promoted can help advise them on that.” (DDD 7) “[I] f you’re a clinician teacher, clinician researcher, you’re surrounded by so many people on that path at different stages that there are tons of role models to follow… by osmosis, [you] get some mentorship… But when you go down, the CQIs…there is no osmosis.” (VC 2) “So your career doesn’t peak until you get to a point where you may be too far gone, right, you may be late 60, 70 and your career just peaking, finishing the peak.” (VC 3) “So there has to be that kind of partnership going on. We have to promote that partnership… And I think that’s what’s helping people. I think that’s where mentoring is coming in.” (VC 3) “I think in research and the CQI, a lot of the help that a mentor can give is around the network, the connection because so much of that work is related to getting help from somebody else, like connecting people.” (DDD 7)
• Social factors	“[R] ace and gender may be important and for some people not at all. So, we shouldn’t assume one way or the other, we should just have the opportunity for those things to be in the matrix if people want them to be in the matrix.” (DDD 5) “I think …an issue for women…is around sponsorship…I think has been an issue and I think it’s a bigger issue for women than it is for men in that there’s less sponsorship.” (DDD 2) “Like there are a lot of areas where women would benefit from specific mentorship about what to do and how to succeed with a family.” (DDD 10) “There’s going to be benefits to finding a culturally convergent peer: someone who has dealt with a lot of the same barriers and inequities of navigating a hostile or potentially a hostile environment…and sharing that knowledge with someone who is treading that path for the first time.” (DDD 11) “I go back and forth on this a little bit. I see the value clearly because there are circumstances in which… you always want to make the mentee comfortable, otherwise the relationship doesn’t work. They need to be able to confide in or bring up topics that they feel would be challenging to talk about with others.” (DDD 8)
***A thoughtful approach towards promoting faculty engagement to mentorship***	
Systemic and policy changes	“[P]erhaps the CFAR document may need to have some mentorship strength to it. I think as a department, we might create some aspect that makes them describe what mentorship they’ve had, I think that’d be useful as well.” (DDD 12)
Innovative mentorship support resources and initiatives	“So I think the biggest issue would be trying to find more interesting ways and thought provoking ways of getting those messages…I think that to me would be the issue if you’re talking about some faculty development, is finding some really creative ways that people can listen to it and engage with it in a way that doesn’t sound judgemental or pejorative.” (DDD 14)
Faculty social events	“I’m really invested in this idea of the free-range chickens. You put a bunch of people in a room and have them kind of wander around and gradually relationships come from that.” (VC 1) “I think it would be good to know what other people are doing.…So, I think it would…I’d like to see-… All the mentors. Like, the mentors in the other divisions [meet] and see what are people’s experiences, what are people doing to promote mentorship, what have they done, how involved are they, what are they actually doing, or are they doing anything.” (MF 1)
Mentorship incentives	“Promotion on mentorship… that would really be quite something…that would be wonderful to see…that mentorship could be something you can promote upon. We’ve really seen change at the university over the past 10 years, 5 years, so it’d be nice to see it on mentorship. There’s no reason why it shouldn’t.” (DDD 13) “Yeah, I mean section three are always challenging to get. If the mentee provides feedback to the mentor and the mentor considers it, section three would be quite attractive to people.” (DDD 13)

Overall, building upon and expanding a pre-existing mentorship culture involves scaling-up mentorship processes transparently and flexibly to meet the unique needs of faculty. Proper prioritization and incentivization is needed to engage faculty in mentorship and thereby expanding the mentorship culture.

#### Application of the Social Ecological Model

Cross-categorization of study findings into levels (relationship, divisional/departmental, academic medicine) and components (expectations, mentor identification, relationship maintenance, mentor recognition, faculty development, measurement) are indicated in Table 5. Looking across the table by rows allowed us to identify level-specific interventions (i.e. what matters for each level), while looking down the table by columns allowed us to identify interventions that target specific components (i.e. what is required to maintain a relationship). Finally, this categorization allowed us to identify leverage points; specifically, having established metrics (mentorship and program metrics) would enable clear expectations, promote mentor identification (e.g. tabulate relationships) and relationship maintenance (e.g. measure relationship quality), enable mentor recognition (e.g. awards) and facilitate faculty development (e.g. outline objectives and outcomes for curriculum). Similarly, mentor recognition was identified as a leverage point as it can facilitate mentor identification, relationship maintenance, and motivation for faculty development.

**Table 5:**
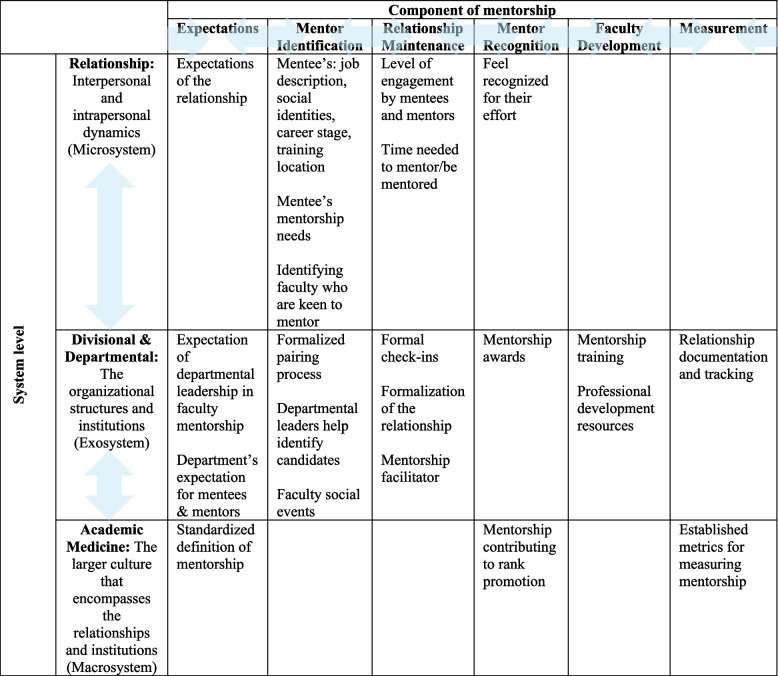
Application of the Social Ecological Model (SEM) highlighting five levels and six components

Application of the Social Ecological Model (SEM) to the study findings highlighting three levels (relationship, divisional & departmental, and academic medicine) and six components (expectations, faculty development, mentor identification, relationship maintenance, mentor recognition, and measurement). The levels are interconnected as are the various components, with interactions being reciprocal thereby increasing the impact of each level and component and creating more flexibility during mentorship program development

## Discussion

While departmental leadership believed a mentorship culture was valued by the department, additional strategies were outlined to build-upon the current mentorship culture, including scaling up pre-existing mentorship processes and creating incentives for participation to promote greater faculty engagement.

Mentorship processes and needs are influenced by mentor-mentee relationships, the nature of the institution, and the current landscape of academic medicine. Application of the SEM model to our findings (Table 5) revealed two components that could be high leverage points (places within a system where small changes can substantially effect the entire system) [22]: tools to measure mentorship outcomes and systems of mentor recognition.

Metrics can outline the department’s mentorship expectations and consequently faculty development initiatives can be implemented in accordance with these expectations. A similar notion was conveyed by Chi et al.: metrics can establish best mentorship practices and support quality improvement [23]. While our findings and the literature [24, 25] outline the importance of evaluating the quality of relationships and documenting mentor-mentee activities, it was unclear how best to evaluate mentorship quality, though participants suggested using tangible (e.g. number of publications) and internal measures of success (e.g. mentee satisfaction), which aligns with the literature [2, 25–27]. However, consideration of the multi-level nature of SEM provides a useful framework. To encapsulate the complexity of faculty mentorship, we created a multilevel evaluation framework from the lens of SEM, as follows. While typically mentorship evaluations have focused on the individual level, for example, mentee satisfaction [2, 27], consideration of other levels [23] broadens metrics to include: tracking the number and diversity of available mentors; the existence of mentor training programs; and the proportion of mentees becoming mentors [23]. Moreover, mentorship metrics are temporal, as the metrics used are in-part dependent on the program’s maturity [23]. Newer programs may focus on short-term outcomes such as relationship quality, while over time, long-term results like career advancement may be emphasized [23]. Overall, using multilevel metrics that measures tangible and internal outcomes while accounting for the maturity of the mentorship program may be an effective approach to capturing the complexity of faculty mentorship.

Recognizing mentors can incentivize the development and maintenance of mentoring relationships, and sustain a mentorship culture. Formalized recognition for mentorship can increase morale and willingness to participate in mentorship, promote career advancement and satisfaction [28, 29], and in doing so, retain diverse and skilled mentors [28, 30–32]. An SEM lens helped us employ a multilevel framework strategy for mentor recognition. We identified that mentor recognition can occur not only at the divisional/departmental level (awards) but also at the academic medicine level (promotion) [28, 30, 31]. Thus, strategies for recognition can focus on supporting the mentor through career advancement (e.g. mentorship contributing to promotions), acknowledgement of their mentoring efforts through public social platforms (e.g. website), and providing awards and stipends (e.g. renumeration, trophy/plaque) [28, 30, 31, 33].

Our study has limitations. First, it includes perspectives of leadership at a single academic site which may limit generalizability. The leverage points we outlined could have been influenced by our large department. Institutions with smaller departments may have different dynamics, and thus, different leverage points when developing a mentorship program. Additionally, participants may have experienced a social desirability bias, a tendency to provide responses that are socially acceptable and viewed favorably; this bias can be difficult to control for in qualitative studies [34]. Our study also has several strengths including the size of the academic department, ascertaining leadership perspective, and novel application of SEM.

Future research can explore the perspectives of departmental administration and faculty members on mentorship within the department. Furthermore, academic institutions can create a mentorship program using the lens of SEM and evaluate its impact.

## Conclusion

In this study, we examined a medicine department’s faculty mentorship program from a departmental leadership perspective and determined the crucial program components. We then outlined a novel approach to developing/enhancing a faculty mentorship program using the SEM model, which accounts for multilevel factors that may influence the success of mentorship. Through the lens of SEM, we determined that mentorship metrics and mentor recognition are potential leverage points in a mentorship program, and we provided a multilevel framework with actionable strategies to implement these leverage points that synergizes with other program components. Our findings can foster the development of meaningful mentoring relationships within an institution and promote faculty success.

### Supplementary Information

**Supplementary Material 1.**

**Supplementary Material 2.**

## Data Availability

The datasets used and/or analyzed during the current study are available from the corresponding author on reasonable request.

## Abbreviations

DDD: Department Division Director
SEM: Social Ecological Model
