# Supplementary material for: Optimizing a mentorship program from the perspective of academic medicine leadership – a qualitative study
Source: BMC Med Educ. 2024 May 14;24:530. doi: 10.1186/s12909-024-05463-6 (PMC11092246; doi:10.1186/s12909-024-05463-6)
Supplement: Supplementary file 1 — Supplementary Material 1. [file 12909_2024_5463_MOESM1_ESM.docx]

Standards for Reporting Qualitative Research (SRQR)*

<http://www.equator-network.org/reporting-guidelines/srqr/>

Title and abstract

Page/line no(s).

| **Title** - Concise description of the nature and topic of the study Identifying the study as qualitative or indicating the approach (e.g., ethnography, grounded theory) or data collection methods (e.g., interview, focus group) is recommended | Page 1 – Title indicates a qualitative study. |
| --- | --- |
| **Abstract** - Summary of key elements of the study using the abstract format of the intended publication; typically includes background, purpose, methods, results, and conclusions | Pages 3-4 – Abstract includes background, purpose, methods, results, and conclusions. |

Introduction

| **Problem formulation** - Description and significance of the problem/phenomenon studied; review of relevant theory and empirical work; problem statement | Pages 5-6 – Describes the significance of the problem/phenomenon studied: Understanding how to create an effective landscape for mentorship in academic medicine in order to facilitate faculty success.  Problem statement: Few studies have explored the perspective of departmental leadership on the crucial factors for an effective mentorship program. Their perspective is valuable because it can provide a more macroscopic and comprehensive understanding of mentorship and how to enhance its effectiveness. |
| --- | --- |
| **Purpose or research questio**n - Purpose of the study and specific objectives or questions | Page 6 – Research question/purpose: To explore the current departmental mentorship processes and to elucidate the crucial components of an effective mentorship program in a medicine department from the perspective of departmental leadership. |

Methods

| **Qualitative approach and research paradigm** - Qualitative approach (e.g., ethnography, grounded theory, case study, phenomenology, narrative research) and guiding theory if appropriate; identifying the research paradigm (e.g., postpositivist, constructivist/ interpretivist) is also recommended; rationale** | Page 7 – Qualitative approach: Qualitative description method. |
| --- | --- |
| **Researcher characteristics and reflexivity** - Researchers’ characteristics that may influence the research, including personal attributes, qualifications/experience, relationship with participants, assumptions, and/or presuppositions; potential or actual interaction between researchers’ characteristics and the research questions, approach, methods, results, and/or transferability | Page 9 – “Data analysis and role of theory” section contains information about researchers’ qualifications/experience and the method of interaction between researchers’ characteristics. |
| **Context** - Setting/site and salient contextual factors; rationale** | Page 7-8 – “Setting and participant” and Description of the Faculty Mentorship Program” sections contain the relevant information. |
| **Sampling strategy** - How and why research participants, documents, or events were selected; criteria for deciding when no further sampling was necessary (e.g., sampling saturation); rationale** | Page 5-6 – Outlines why departmental leaders were selected.  Pages 8 – “Recruitment” section contains information about sampling strategies.  Page 9 – “Data analysis and role of theory” section describes criteria for data saturation. |
| **Ethical issues pertaining to human subjects** - Documentation of approval by an appropriate ethics review board and participant consent, or explanation for lack thereof; other confidentiality and data security issues | Pages 7 and 22 |
| **Data collection methods** - Types of data collected; details of data collection procedures including (as appropriate) start and stop dates of data collection and analysis, iterative process, triangulation of sources/methods, and modification of procedures in response to evolving study findings; rationale** | Pages 7 – Type of data collected: interviews.  Page 3 – Start date: April 2021, End date: December 2021  Page 9 – “Data analysis and role of theory” section outlines method of analysis: independent coders, triangulation demonstrated through each researcher having unique experiences and backgrounds, and coding discrepancies resolved through routine research meetings. |
| **Data collection instruments and technologies** - Description of instruments (e.g., interview guides, questionnaires) and devices (e.g., audio recorders) used for data collection; if/how the instrument(s) changed over the course of the study | Page 8 – “Data collection” section contains information about instruments and devices.  Instrument: interview guide  Device: Zoom audio recording |
| **Units of study** - Number and relevant characteristics of participants, documents, or events included in the study; level of participation (could be reported in results) | Page 9 – “Characteristics of participants” section states the number of participants.  See Table 1 for characteristics of participants. |
| **Data processing** - Methods for processing data prior to and during analysis, including transcription, data entry, data management and security, verification of data integrity, data coding, and anonymization/de-identification of excerpts | Page 8-9 – “Data collection” section contains information about methods of processing the data. |
| **Data analysis** - Process by which inferences, themes, etc., were identified and developed, including the researchers involved in data analysis; usually references a specific paradigm or approach; rationale** | Pages 9 – “Data analysis and role of theory” section contains the relevant information. |
| **Techniques to enhance trustworthiness** - Techniques to enhance trustworthiness and credibility of data analysis (e.g., member checking, audit trail, triangulation); rationale** | Pages 9 – “Data analysis and role of theory” section describes techniques to enhance trustworthiness.  Independent coders and routine research meetings to resolve discrepancies. Each researcher had unique backgrounds and experiences. Field notes were made, and coding framework was developed through an iterative process. |

Results/findings

| **Synthesis and interpretation** - Main findings (e.g., interpretations, inferences, and themes); might include development of a theory or model, or integration with prior research or theory | Pages 9-19 – “Results” section contains main findings: themes and application of Social Ecological Model. |
| --- | --- |
| **Links to empirical data** - Evidence (e.g., quotes, field notes, text excerpts, photographs) to substantiate analytic findings | See Tables 2-5. |

Discussion

| **Integration with prior work, implications, transferability, and contribution(s) to the field -** Short summary of main findings; explanation of how findings and conclusions connect to, support, elaborate on, or challenge conclusions of earlier scholarship; discussion of scope of application/generalizability; identification of unique contribution(s) to scholarship in a discipline or field | Page 19 – “Discussion” section, paragraphs 1 and 2 contains summary of main findings.  Pages 19-21 – “Discussion” section, paragraphs 3 and 4 explain how findings and conclusions relate to earlier scholarship.  Page 21-22 – “Conclusion” section describes unique contribution. |
| --- | --- |
| **Limitations** - Trustworthiness and limitations of findings | Page 21 – “Discussion” section, paragraph 5. |

Other

| **Conflicts of interest** - Potential sources of influence or perceived influence on study conduct and conclusions; how these were managed | Page 22 – None. |
| --- | --- |
| **Funding** - Sources of funding and other support; role of funders in data collection, interpretation, and reporting | Page 22-23 – This study was funded by the Royal College of Physicians and Surgeons of Canada Medical Education Research Grant. |

*The authors created the SRQR by searching the literature to identify guidelines, reporting standards, and critical appraisal criteria for qualitative research; reviewing the reference lists of retrieved sources; and contacting experts to gain feedback. The SRQR aims to improve the transparency of all aspects of qualitative research by providing clear standards for reporting qualitative research.

| **The rationale should briefly discuss the justification for choosing that theory, approach, method, or technique rather than other options available, the assumptions and limitations implicit in those choices, and how those choices influence study conclusions and  transferability. As appropriate, the rationale for several items might be discussed together. |
| --- |
| **Reference:**  O'Brien BC, Harris IB, Beckman TJ, Reed DA, Cook DA. Standards for reporting qualitative research: a synthesis of recommendations. *Acad Med*. Sep 2014;89(9):1245-51. doi:10.1097/acm.0000000000000388 |
|  |
