# Supplementary material for: Optimizing a mentorship program from the perspective of academic medicine leadership – a qualitative study
Source: BMC Med Educ. 2024 May 14;24:530. doi: 10.1186/s12909-024-05463-6 (PMC11092246; doi:10.1186/s12909-024-05463-6)
Supplement: Supplementary file 2 — Supplementary Material 2. [file 12909_2024_5463_MOESM2_ESM.docx]

**Additional file 2: Semi-structured interview guide**

| **Topic** | **Opening Questions** | **Prompts** |
| --- | --- | --- |
| **Challenge** | What are the main challenges to mentorship in your Division? | Finding mentor  Time to mentor |
| **Prevalence** | What proportion of the Division has mentors? | Not tracked but likely everyone has informal mentor (guidance, advice, sharing etc) |
|  | How do you track this? | Not tracked |
|  |  |  |
| **Process** | | |
| Pairing | Other than the formal relationship assigned at appointment, how have faculty been paired with mentors? | How long do these relationships persist?  Are these relationships tracked? |
|  | Whose responsibility is it ? | DDD?  PIC?  Local Division director  Anyone else?  Do you have terms of reference? For Divisonal exec? Mentorship? |
|  | Who initiates the process? | Faculty member?  Leadership? |
|  | What have been the activities of the mentorship facilitator? | Are they still active?  Were they effective? |
|  | How do you identify potential mentors? | What do you think of creating a database of willing people based on interests and roles |
|  |  |  |
| **Need** | Are their different faculty groups that need more/different mentoring? | Mid-career? Late-career?  Job description?  Academic rank?  Race/ethnicity?  Gender? |
|  | Late | Given the career stage and experience of later career faculty, how might you enable mentoring of this population? |
|  |  |  |
| **Incentives** |  |  |
| Divisional mentorship award | Is there one? | Did it work? |
|  | Will you make one? | How frequent? |
|  | What will it entail? | Plaque, $? |
|  | What is the process for selecting an awardee? | Selection committee?  How? Nominated? Selection criteria?  Would you be interested in adopting these mentorship award committee criteria? |
|  |  |  |
| Are there other incentives you can think of? | For mentor | Formalization of relationship?  MOC credits? |
|  | For mentee? |  |
|  |  |  |
| **Resources used/Documentation of mentorship** | | |
| What resources or documents do you use for mentoring or to document mentoring? | Mentorship checklist |  |
|  | Academic planning document |  |
|  | Annual Review/Report |  |
|  |  |  |
| **Metrics** | | |
| What are important metrics related to mentoring? | Relationship quality |  |
|  | How many are mentored? |  |
|  | How many mentor? |  |
|  | Productivity? |  |
|  |  |  |
| **Other related mentorship activities** | | |
| Other roles | What are other roles of a mentor? | Sponsorship? |
|  |  | awards nomination? |
|  |  | Allyship ? |
|  |  | Advocacy? |
|  | How are the following issues addressed in your Division? | parity equity (daily work, e.g. clinic room), treatment in work environment |
|  |  | Gender |
|  |  | Ethnicity |
|  |  | Wellness, burnout |
